# High Density Body Surface Potential Mapping with Conducting Polymer‐Eutectogel Electrode Arrays for ECG imaging

**DOI:** 10.1002/advs.202301176

**Published:** 2023-05-18

**Authors:** Ruben Ruiz‐Mateos Serrano, Santiago Velasco‐Bosom, Antonio Dominguez‐Alfaro, Matias L. Picchio, Daniele Mantione, David Mecerreyes, George G. Malliaras

**Affiliations:** ^1^ Electrical Engineering Division University of Cambridge Cambridge CB3 0FA UK; ^2^ POLYMAT University of the Basque Country UPV/EHU Avda. Tolosa 72 Donostia‐San Sebastian Gipuzkoa 20018 Spain; ^3^ IKERBASQUE Basque Foundation for Science Bilbao 48009 Spain

**Keywords:** body surface potential maps, conducting polymer, electrocardiography, non‐invasive imaging

## Abstract

Electrocardiography imaging (ECGi) is a non‐invasive inverse reconstruction procedure which employs body surface potential maps (BSPM) obtained from surface electrode array measurements to improve the spatial resolution and interpretability of conventional electrocardiography (ECG) for the diagnosis of cardiac dysfunction. ECGi currently lacks precision, which has prevented its adoption in clinical setups. The introduction of high‐density electrode arrays could increase ECGi reconstruction accuracy but is not attempted before due to manufacturing and processing limitations. Advances in multiple fields have now enabled the implementation of such arrays which poses questions on optimal array design parameters for ECGi. In this work, a novel conducting polymer electrode manufacturing process on flexible substrates is proposed to achieve high‐density, mm‐sized, conformable, long‐term, and easily attachable electrode arrays for BSPM with parameters optimally selected for ECGi applications. Temporal, spectral, and correlation analysis are performed on a prototype array demonstrating the validity of the chosen parameters and the feasibility of high‐density BSPM, paving the way for ECGi devices fit for clinical application.

## Graphical abstract

A high‐density conducting polymer electrode array for body surface potential mapping (BSPM) is proposed, with parameters optimally selected for electrocardiographic imaging (ECGi) applications. Temporal, spectral, and correlation analyses performed on prototype arrays confirm the optimal set of parameters and demonstrate the feasibility of high‐density BSPM, paving the way for ECGi devices fit for clinical application.

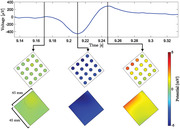

## 1 Introduction

Cardiovascular diseases (CVD) account for ≈32% of global mortality, making it the undisputed leading cause of death worldwide. It is also a major contributor to disability and healthcare system saturation, especially in low‐ and middle‐income countries.^[^ ^1^ ^]^ Most CVD can be prevented by tackling behavioral risk factors at an early stage and by administering drugs.^[^ ^2^, ^3^, ^4^ ^]^ Electrocardiography (ECG) is a well‐established technique for cardiac dysfunction diagnosis which has been employed for decades. However, ECG has been shown to lack spatial resolution and cannot be employed to accurately diagnose complex heart conditions, such as acute ischemia, infarction, or coronary artery disease, forcing clinicians to resort to invasive procedures.^[^ ^5^, ^6^, ^7^, ^8^, ^9^, ^10^ ^]^ Recent work has focused on the development of improved catheter and flexible electrode array technologies able to map the surface of the cardiac muscle wall with very high spatial resolution.^[^ ^11^, ^12^, ^13^, ^14^ ^]^ Multifunctional membranes able to both record and stimulate across the entire epicardium have also been reported as a means to minimize insertion requirements.^[^ ^15^ ^]^ Body surface potential mapping (BSPM) has been proposed as an alternative technique to achieve non‐invasive and continuous cardiac electrical activity measurements with high spatial resolution.^[^ ^16^, ^17^, ^18^ ^]^ Despite its advantages, BSPM has not become a routinely employed clinical method, principally due to lack of interpretability of surface potentials, which do not resemble epicardial potentials.^[^ ^19^ ^]^

Inverse procedures which reconstruct bioelectric sources from BSPM, known as ECG imaging (ECGi) techniques, have been proposed to overcome interpretability limitations.^[^ ^20^, ^21^, ^22^, ^23^, ^24^, ^25^, ^26^, ^27^, ^28^, ^29^, ^30^, ^31^, ^32^, ^33^, ^34^, ^35^ ^]^ ECGi algorithms augment raw BSPM data with knowledge of the location of electrodes and additional anatomical information. They then apply electric field propagation equations to yield multiple valid solutions to the inverse problem. The challenge lies in obtaining a solution which is not only mathematically correct but biologically plausible by choosing the right set of conditions. As a consequence of the ill‐posed nature of the inverse problem, ECGi techniques lack sufficient precision to be effectively employed in clinical setups.^[^ ^36^, ^37^ ^]^

BSPM are limited to a fixed number of sampling points due to practical restrictions. Commercial Ag/AgCl electrodes employed in clinical setups to measure ECG need to be positioned individually at exact locations, are poorly conformable to the surface of the skin, occupy relatively large areas, and need to be replaced after a certain number of hours. Furthermore, recording devices limit the total number of channels and maximum signal storage. BSPM with sub‐optimal sampling can be sufficient for clinical diagnosis through human inspection but have a significant impact on the conditioning of ECGi algorithms and hence their accuracy. Much work has focused on minimizing error by optimizing electrode positioning, but results are still insufficient.^[^ ^38^, ^39^, ^40^, ^41^, ^42^, ^43^, ^44^, ^45^, ^46^, ^47^ ^]^ State‐of‐the‐art BSPM arrays reported in the literature and industry (Medtronic CardioInsight) present a maximum number of electrodes ranging from 100 to 300 and achieve reconstruction accuracies between 60% and 80%.^[^ ^48^ ^]^

Electronics have advanced considerably in the past 2 decades and new sampling techniques have been developed which can overcome the aforementioned storage and channel restrictions.^[^ ^49^, ^50^, ^51^ ^]^ Furthermore, additive manufacturing techniques are giving rise to freeform 3D structural electronics which are being used to improve circuit integration in wearable devices.^[^ ^52^ ^]^ Flexible, large‐area, on‐skin electrode technologies for health monitoring have advanced considerably in the past decade, enabling the fabrication of easily attachable, mm‐sized electrode arrays with increased conformability.^[^ ^53^, ^54^, ^55^ ^]^ Coating materials employing conducting polymers, such as poly(3,4‐ethylenedioxythiophene):poly(styrene sulfonate) (PEDOT:PSS), have been engineered to integrate both electronic and ionic conductivity properties in a biocompatible manner. Conducting polymers behave as ideal volumetric capacitors and can increase the effective contact area at the electrode–skin interface which allows for electrode designs with reduced area.^[^ ^56^, ^57^, ^58^ ^]^ Despite the advantages conducting polymer coatings offer, electrodes still present high impedance and poor contact with the skin. Commercially available ionically conductive pastes (CP) are typically employed to improve signal quality during measurements, however, they dehydrate over time.^[^ ^59^ ^]^ New generations of additives based on deep eutectic solvents (DES) have been developed as inexpensive alternatives to CP which ionically bind with conducting polymers to retain enhanced conductivity.^[^ ^60^, ^61^, ^62^, ^63^ ^]^ PEDOT doped with lignin sulfonate (PEDOT:LS) in combination with DES has been shown to yield improved ionic and electronic conductivity.^[^ ^64^ ^]^ Furthermore, combining PEDOT:LS/DES with gelatine has conceived a new type of mixed conducting eutectogels (ETG) which have been shown to have excellent tridimensionality and conformability when employed as coating for electrode recordings.^[^ ^65^, ^66^ ^]^

These new advances in multiple disciplines allow for the development of BSPM arrays with unprecedented spatial resolution and pose questions concerning array design parameters such as electrode shape, area, and inter‐electrode distance (IED). Some of these questions have been answered for high‐density electromyography (EMG) and specific pathological condition detection in BSPM but have not been answered for ECGi applications.^[^ ^67^, ^68^ ^]^

In this paper, a novel electrode fabrication process with flexible substrates based on ETG electrode coating is presented and employed to achieve high‐density, conformable, long‐term, and easily attachable electrode arrays for BSPM with parameters optimally selected for ECGi applications. Conventional ECG measurements from commercial Ag/AgCl and fabricated electrodes with four times less area are compared, showing similar signal‐to‐noise ratio performance. Simultaneous electrode array measurements from fabricated devices demonstrate that distinct ECG signals can be obtained from each electrode, yielding temporal and spatial information such as direction of propagation and speed, spectral information, and correlations. The recorded BSPM are interpolated without aliasing distortions and results are presented which confirm the validity of the optimal array design parameter selection.

## 2 Results and Discussion

**Figure 1:**
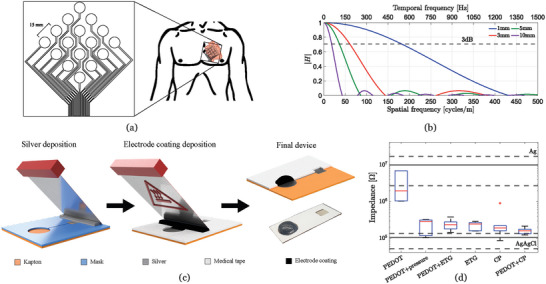
a) Sixteen electrode array design with IED of 15 mm, b) transfer function of circular electrode design as a function of circle radius. Temporal and spatial frequencies are shown for a conduction velocity of 3 m s^−1^ (based on^[^ ^68^ ^]^), c) schematic (not to scale) of the electrode fabrication process: silver paste is blade‐coated onto Kapton using a temporary mask, then double‐sided medical‐grade tape is attached and employed as a mask to blade‐coat the electrode coating at a working temperature of 120 °C. Cross section of electrode and insulation shown as the final image, and d) impedance measurements at 50 Hz for different coating materials, with printed Ag and commercial, gel‐assisted Ag/AgCl electrodes presented as reference (three measurements are taken on six devices with the same coating).

A 16 electrode array has been fabricated as shown in **Figure** **1**a. The electrodes are circular, have a radius of 5 mm and an IED of 15 mm. Electrode shape, size, and IED parameters were calculated based on physiological information and mathematical analysis:

First, the shape of the electrodes is chosen as a consequence of the direction of propagation of the signal of interest. In EMG applications, where signals travel in a single direction along the muscle fibers, it is common to employ square or rectangular electrodes and place them so that the signal of interest crosses the shortest distance from one end of the electrode to the other. This ensures electrode area is maximized, which improves signal‐to‐noise ratio by reducing overall electrode impedance, while avoiding a decrease in spatial resolution.^[^ ^68^ ^]^ Since ECG signals propagate in various directions during a heartbeat, a radially symmetric circular shape is preferred.

Second, the IED is calculated by finding a compromise between spatial resolution and device fabrication complexity. Increasing the density of electrodes improves BSPM quality but also requires thinner wiring and lower separation between electrodes. In order to maximize fabrication yield, the optimal IED should be the largest possible distance which ensures there is no loss of information. In order to avoid errors or distortions due to lack of samples, signals must be measured at twice the highest frequency reached, as stated by the Nyquist theorem.^[^ ^69^ ^]^ The frequency (*f*) characteristics of ECG signals are standardized in clinical settings, with limits between 0.5 and 100 Hz,^[^ ^70^ ^]^ that is, a minimum sampling of 200 Hz. The minimum IED required can be calculated if the conduction velocity (*CV*) of action potentials across tissue is assumed constant during a cardiac cycle. This is common in physiology, where the *CV* is approximated to 3–5 m s^−1^.^[^ ^71^ ^]^ The IED is given by IED = CV/*f*. For a worst‐case scenario of CV = 3 m s^−1^, an IED of 15 mm is optimal.

Last, the optimal electrode area is calculated by considering the trade‐off between spatial resolution and signal quality introduced by a single circular electrode. The signals travelling under the surface of an electrode are averaged and, as a consequence, fast variations (i.e., higher‐frequency components) of the signal are lost, producing a low‐pass filtering effect. The impulse response of a circular electrode h(*x*, *y*) can be obtained purely from geometry^[^ ^72^ ^]^

(1) $$h\left(x,y\right)\left\{\begin{array}{cc} 1/\pi r^{2} & if\;x^{2}+y^{2}\leq r^{2}\\ 0 & otherwise \end{array}\right.$$

where *x* and *y* represent the horizontal and vertical position with respect to the center of the circle respectively and *r* indicates the radius of the circle. The Fourier transform of Equation (1) yields the transfer function of a circular electrode which relates the attenuation effect to signal frequency

(2) $$H\left(s_{x},s_{y};r\right)=\frac{2J_{1}\left(2\pi r\sqrt{s_{x}^{2}+s_{y}^{2}}\right)}{2\pi r\sqrt{s_{x}^{2}+s_{y}^{2}}}$$

where *s_x_* and *s_y_* represent spatial sampling (in cycles/m) along the *x* and *y* directions, respectively, and *J*_1_ indicates the Bessel function of first kind and order. Equation (2) is depicted in Figure 1b for different values of *r* and shows how increasing the radius of an electrode augments the averaging effect. It can be inferred that if the electrode radius is too large the IED is constrained and if it is too small the electrode impedance increases, yielding suboptimal signal quality. In accordance with this, for the range of frequencies of interest (i.e., 0.5–100 Hz), a compromise on this trade‐off is achieved for a radius of 5 mm, the maximum electrode size which will not attenuate ECG information beyond a 3 dB point.

The geometrical parameters hereby presented have been calculated based on the spatiotemporal properties of cardiac action potentials propagating across the skin and not on specific anatomical features. This implies that these design choices are valid for subjects with different chest or heart size. The final recorded 3D BSPM will differ between different subjects and the mechanical placement of the electrode vest will need to be tailored to each body shape. However, the IED, size, and shape of the electrodes in these vests should be the same over the entire torso to ensure no loss of information occurs. The specific positioning of the electrode array patch presented in Figure 1a is non‐critical for parameter validation purposes since BSPM information is present throughout the entire surface of the torso. Variability in heart rate due to age or pathological conditions such as tachycardia are also accounted for in the presented design since a maximum bandwidth of 100 Hz (6000 bpm) has been assumed.

The electrode array fabrication process is described in Figure 1c, with details given in the Experimental Section (for a visual description of the process, Figure S1, Supporting Information). A 70 µm silicon‐based polyester tape is patterned with the shape of electrodes and metal tracks by laser and attached to a 50 µm thick polyimide film (Kapton) substrate to act as a mask. The tape is chosen for its thermal stability, minimal adhesive residue, and simple peel‐off. Silver nanoparticle paste is blade‐coated and cured to remove solvents. The silicon tape is subsequently removed and replaced by a 120 µm thick double‐sided medical‐grade tape patterned by laser to cover the metal tracks only. The medical tape mask achieves insulation of the wires in contact with the skin and procures adhesion to the entire array once the top liner is removed. It was chosen specifically for its skin compatibility and long‐term adhesion. The gaps generated by the tape around the surface of the electrodes are employed to deposit the coating material by blade‐coating it at a working temperature of 120 °C. After allowing some time for cool‐down, the final device is ready to be applied to the skin by removing the medical tape liner and has an overall thickness of 170 µm with electrode areas protruding ≈1 mm on average. The flexibility of the Kapton substrate and the medical tape offer conformability and adhesiveness to the array while the electrode area protrusions improve skin/electrode interfacing and recorded signal quality. Connectivity to recording electronics is achieved through an FFC/FPC jumper cable, wire‐bonded to the array with anisotropic conducting film (ACF).

The selection of coating material is based on comparative impedance measurements of single electrodes as shown in Figure 1d (for impedance measurements swept between 0.1 and 100 000 Hz, Figure S2, Supporting Information, more detailed coating material description in the Experimental Section). Bare Ag electrodes without coating and commercial Ag/AgCl gel‐assisted electrodes are presented as baselines for worst and best performance, respectively. Drop‐casted PEDOT:PSS reduces the impedance of bare electrodes but exhibits a poor performance compared to commercial electrodes. When external pressure is applied to the electrodes, a significant (*P* = 0.3416) improvement in performance is observed which indicates the high impedance of PEDOT:PSS electrodes is mostly due to weak skin/electrode contact. When commercial CP is applied to both bare and PEDOT:PSS coated electrodes, a significant decrease (*P* = 0.093, *P* = 0.3407, respectively) in impedance is also observed. This is explained by the increase in ionic transfer procured by the gel, consequence of improved mechanical contact and reduced transfer resistance. Despite their improved performance, electrode coatings with CP cannot be applied in long‐term measurements due to volatilization, as previously mentioned. ETG was analyzed independently and in addition to PEDOT:PSS coating and showed similar impedance and adhesion to electrodes with CP or externally applied pressure (adhesion strength of 80 kPa and adhesion energy of 33 J m^−1[^ ^66^ ^]^). ETG‐only electrodes exhibit slightly reduced impedance compared to ETG with coated PEDOT:PSS electrodes and are thus selected as optimal array coating material. ETG‐coated electrodes show similar impedance values to those previously reported for conducting polymer electrodes.^[^ ^66^, ^73^, ^74^, ^75^, ^76^, ^77^, ^78^, ^79^ ^]^

**Figure 2:**
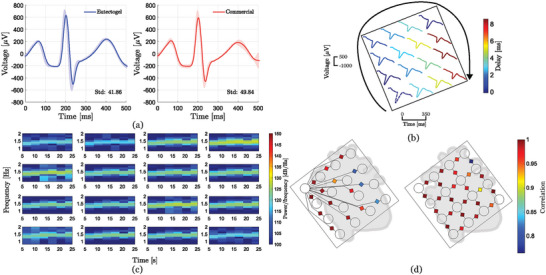
a) Time‐independent ensemble average of ECG peaks generated from 30 s (≈43 peaks) lead I measurements from eutectogel and commercial Ag/AgCl electrodes. The average standard deviation is included, b) single cardiac potential measured by different electrodes in the test array with color scale indicating time delays with respect to the bottom electrode (dashed line), c) spectrograms of individual electrodes in the test array for a 30 s measurement, and d) Correlation between signals recorded at different electrodes as a function of distance from the left‐most electrode (left) and between neighboring electrodes (right).

Results from a conventional three electrode ECG recording setup (Figure S3, Supporting Information) comparing ETG and commercial electrodes are presented in the ensemble average plots in **Figure** **2**a, which were generated from 30 s lead I recordings. The measurements obtained from both devices offer very similar waveforms, both in shape (including P wave, QRS complex, and T wave), and in magnitude (reaching maximum amplitude values of ≈750 µV). The similarity in the period of the measured signals indicates the pulse of the subject remained at a constant value of ≈85 bpm (i.e., a period of ≈700 ms) for both experiments. The amplitude variability and the average standard deviation are comparable between both signals, despite the fact that commercial electrodes occupy four times greater area than ETG electrodes. ECG SNR values from previously reported conducting polymer electrodes are comparable to the values obtained in this work.^[^ ^66^, ^73^, ^74^, ^75^, ^76^, ^77^, ^78^, ^79^ ^]^ Figure 2b depicts a single cardiac potential measured by different electrodes in the test array (Figure S4, Supporting Information). The delay between signals with respect to the bottom electrode (dashed line) is represented by a color map. Analysis of the spatial distribution of the delays allows the inference of information concerning the speed of propagation of action potentials, ≈4.4 m s^−1^, in accordance with standard CV values (3–5 m s^−1[^ ^54^ ^]^), thus validating the proposed electrode design. It also offers information on the direction of propagation of body surface potentials which, unlike epicardial potentials, spread peripherally following a circular trajectory with a maximum delay of ≈8 ms, as can be observed from the measurements in Figure 2b.

The spectrograms generated by all electrodes in the array for 25 s recordings are depicted in Figure 2c. Initial transients from the first five s of the recording are omitted for clarity. The spectrograms offer insight on the temporal evolution of the heart rate of the subject during the trial, which is represented by the peak harmonic trend between 1 and 2 Hz (i.e., 60 to 120 bpm). Channels located at the bottom of the array present slightly larger magnitude values, which reflects the greater strength of ventricular contraction over atrial contraction. Figure 2d represents Pearson coefficient correlation values between electrodes as a function of distance with respect to the left‐most electrode and between neighboring electrodes. Correlation values are above 0.7 for all electrodes, indicating certain information overlap. As expected, the correlation decreases as a function of distance and also describes the direction of propagation of the action potentials, in accordance with Figure 2b, since the three right‐most electrodes present a significantly lower correlation than the rest. Neighboring electrodes present similar correlations except for the three right‐most electrodes which are highly correlated among themselves but less correlated with their surrounding electrodes.

**Figure 3:**
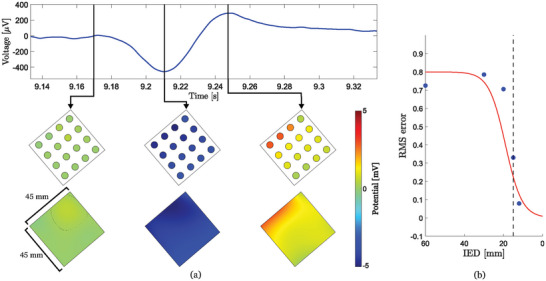
a) Spline interpolation results generated at three distinct stages of a cardiac potential. From left to right: electrically excitable resting state, depolarization, and repolarization. b) RMS error as a function of inter‐electrode distance. The error is calculated between interpolated data obtained from a 10 mm IED array (employed as ground truth) and arrays with different IED acquired through digital undersampling of the ground truth array. Dashed line indicates the derived 15 mm threshold IED, scattered points represent averaged measurements over a hundred time frames and red line depicts an error function fitting.

The individual potential measurements obtained from the test array can be combined into frames describing sections of a BSPM at every point in time during a cardiac cycle (see videos SV1 and SV2, Supporting Information). **Figure** **3**a presents frames generated at three distinct phases during a heartbeat: at an electrically excitable resting state, at a depolarization (contraction) state, and at a repolarization (relaxation) state. The depolarization state is characterized by negative voltage values whereas the repolarization state exhibits a characteristic positive voltage peak produced by ventricular cardiac cells (i.e., the T wave). The sample points obtained from the array are interpolated to achieve continuous BSPM images as shown at the bottom of Figure 3a.

Up to this point, it has been shown how optimal electrode array parameters for ECGi applications can be calculated analytically. The chosen electrode area and shape have been validated by comparing device performance with Ag/AgCl commercial electrodes and presenting results which show how the designed array enables detailed analysis of delays, spectral responses, and correlation. However, the calculated optimal IED remains to be validated practically. If all the assumptions aforementioned were correct, the interpolated images obtained in Figure 3a should not present undersampling distortion errors and should be perfect representations of BSPM.^[^ ^69^ ^]^ In order to validate this hypothesis on real data, a triangular array of 28 electrodes with a radius of 3.5 mm and an IED of 10 mm was fabricated and measured to yield multiple frames of a BSPM (Figure S5, Supporting Information).

Each frame obtained from this array was interpolated to generate a ground truth (GT) BSPM image. This image was undersampled at different rates to generate multiple virtual frames with different IED. These new frames were again interpolated and compared against the GT image, resulting in the RMS error plot in Figure 3b (for further visual description, Figure S6, Supporting Information, further description in the Experimental Section). The data shows that reducing the IED of the triangular array below 15 mm does not contribute significantly to reducing BSPM sampling errors (Figure 3b), confirming the hypothesis.

The results hereby presented demonstrate that advances on large‐area, flexible electronic fabrication methods, in combination with novel conducting polymer coatings, can be employed to overcome the final limiting factor in the development of highly accurate BSPM measurements for ECGi applications. In particular, the silver nanoparticle blade‐coating fabrication method described in this paper has been presented as an inexpensive, scalable, and straightforward technique to make arrays with mm‐size electrodes. The optimal electrode area and IED for ECGi applications have been calculated analytically and validated in practice, offering guidelines for future scaling of complete BSPM recordings. Furthermore, ETG has been shown to provide improved impedance performance with respect to other polymer‐based coatings due to increased mechanical contact at the skin/electrode interface.

The integration of these arrays with state‐of‐the‐art signal processing hardware and machine learning reconstruction techniques paves the way toward the adoption of ECGi devices into clinical settings. In future, these devices could potentially substitute conventional, low spatial resolution 12‐lead ECG in the identification of CVD.^[^ ^80^ ^]^ Furthermore, the high conformability, improved mechanical contact, and impedance performance of the proposed array offers the possibility of expanding its use into different applications for wearable devices, including high‐density EMG, electroencephalography, and fetal movement identification, among others. The use of standalone flexible and stretchable sensing platforms could further improve the long‐term usability of these devices, allowing for a complete integration of device powering, processing, and interfacing.^[^ ^81^ ^]^ Despite the increased conformability Kapton substrates offer to the fabricated electrode arrays, they are still not sufficiently flexible for large‐scale implementations of BSPM covering the entire torso. A detailed investigation on the effects of mechanical deformations (such as stretching or bending) and environmental conditions (such as temperature or moisture) should also be undertaken to study the robustness of the proposed device. Long‐term stability of the electrode array has not been explicitly shown since it is expected to be exclusively dependent on that previously reported for the ETG coating employed, however, further stability studies should be undertaken in future.^[^ ^66^ ^]^ The adhesiveness of the array procured by the layer of medical tape, although long‐lasting, does eventually decay over time. Future work should consider more flexible substrates, such as textiles, which could be attached to the chest or other body parts without a need for adhesive while still maintaining a stable skin/electrode interface.

## 3 Conclusion

In this paper, a novel electrode fabrication process with flexible substrates based on ETG electrode coating polymer coating has been presented and employed to achieve high‐density, conformable, long‐term, and easily attachable electrode arrays for BSPM with parameters optimally selected for ECGi applications. Conventional ECG measurements from commercial Ag/AgCl and ETG electrodes with four times less area have been compared, showing similar signal‐to‐noise ratio performance. Simultaneous electrode array measurements from fabricated devices demonstrate that distinct ECG signals can be obtained from each electrode, yielding temporal and spatial information such as direction of propagation and speed, spectral information, and correlations. The recorded BSPM have been interpolated without aliasing distortions and results have been presented which confirm the validity of the optimal array design parameter selection.

## 4 Experimental Section

### Electrode Fabrication

A silicone release agent‐free blue adhesive plastic tape (SPS, PN:1008R‐6.0) was laser cut to act as a mask for the electrode array and wiped gently with isopropanol to remove debris from the cutting process. Silver nanoparticle paste (Asahi Kagaku, PN:LS453‐6B) was mechanically mixed with a swab before placement at one end of the electrode array mask to manually blade‐coat Ag tracks on a polyimide substrate (Kapton 54×40 mm, 50 µm thick) employing a metal squeegee. Thermal sintering was applied to the paste (80 °C, 60 min) in a conventional oven, according to the manufacturer's specifications. For device insulation and adhesion, double‐side medical‐grade polyethylene tape (3M, PN:1509) was laser cut to fit the pattern of the tracks on the Kapton substrate. The bottom liner was removed and the medical tape was adhered to the substrate, leaving electrode contact area and external connections exposed. ETGs were synthesized by first dispersing PEDOT:LS (2% w/v) powder into the DES (glycerol/choline chloride 2:1 mole ratio), second mixing gelatin (20% w/v) at 90 °C into the PEDOT:LS/DES and finally cooling overnight at 4 °C to promote gelatin triple helix formation based on hydrogen bonds. The produced ETG was blade‐coated on a heating plate at a working temperature of 120 °C by means of a metal squeegee. ACF bonding was applied between the device and a commercial zero insertion force connector (sixteen channel, 1.25 mm pitch) that matched the 500 µm track width.

### Coating Materials

Commercially available PEDOT:PSS (Heraeus Clevios 1 wt%) was mixed (5 wt%) with ethylene glycol (EG), to increase its electronic conductivity. This was followed by the inclusion of 0.1% in weight of dodecyl benzene sulphonic acid, which acts both as a surfactant and as a doping agent for the polymer chain and finally by including 1% in volume of solution of (3‐glycidyloxypropyl)trimethoxy silane, which generates encapsulating covalent bonds around the PEDOT:PSS structure, making it water‐insoluble. The conductive paste employed was purchased (AC cream, Spes Medica S.r.l.) and applied gently on the skin with a swab.

### In Vivo Recordings

All in‐vivo experiments were performed with the approval of the Ethics Committee of the Department of Engineering at the University of Cambridge (06/09/2018, IONBIKE) and after obtaining informed consent from volunteers. The chest of volunteers was prepared by gently wiping ethanol on the skin around the area of interest. The top liner was removed from the device before attaching it to the skin. For impedance measurements, a PGSTAT128N Metrohm AUTOLAB potentiostat was employed, with a single commercial Ag/AgCl electrode (MLA1010B, ADInstruments) acting both as reference and counter electrode. For ECG recordings, a commercial Ag/AgCl electrode was used as reference. All 16 electrodes were recorded simultaneously at a sampling frequency of 30 kHz using a RHS stimulation and recording system from INTAN technologies. The acquired potentials were filtered using a 50 Hz notch filter, and a band‐pass filter with cut‐off frequencies of 0.5 and 100 Hz.

### Statistical Analysis

All ECG signals presented were preprocessed by applying notch filtering at 50 Hz and bandpass filtering between 0.1 and 100 Hz. *P* values for coating material impedance were calculated by means of one‐sample *t*‐tests which were run on MATLAB with 18 samples from each material under comparison (three measurements on six devices with the same coating material). A significance level of 5% was considered sufficient to reject the null hypotheses that the impedance values from both materials came from the same normal distribution. Delays in Figure 2b were calculated by comparing peak potential values between individual electrode recordings and taking an average over the entire duration of a trial. For the RMS error values obtained in Figure 3b, 1000 frames from the triangular array measurements at different periods of the cardiac cycle were interpolated by means of spline interpolation yielding 1000 GT images. Undersampling of these images was achieved by taking the Gaussian average of pixel intensity values surrounding the area that would be covered by electrodes placed at different IED. Final RMS values represented the average of the RMS values calculated for each of the 1000 frames.

## Conflict of Interest

The authors declare no conflict of interest.

## Supporting information

Supporting Information

Supplemental Video 1

Supplemental Video 2

## Data Availability

The data that support the findings of this study are available from the corresponding author upon reasonable request.
