# Supplementary material for: High Density Body Surface Potential Mapping with Conducting Polymer‐Eutectogel Electrode Arrays for ECG imaging
Source: Adv Sci (Weinh). 2023 May 18;11(27):2301176. doi: 10.1002/advs.202301176 (PMC11251564; doi:10.1002/advs.202301176)
Supplement: Supplementary file 1 — Supporting Information [file ADVS-11-2301176-s001.pdf]

ADVANCED SCIENCE
Open Access

Supporting Information

for *Adv. Sci.*, DOI 10.1002/advs.202301176

High Density Body Surface Potential Mapping with Conducting Polymer-Eutectogel Electrode Arrays for ECG imaging

*Ruben Ruiz-Mateos Serrano, Santiago Velasco-Bosom, Antonio Dominguez-Alfaro, Matias L. Picchio, Daniele Mantione, David Mecerreyes and George G. Malliaras**

Supporting information

High Density Body Surface Potential Mapping with Conducting Polymer-Eutectogel Electrode Arrays for ECGi

Ruben Ruiz-Mateos Serrano[1], *Santiago Velasco-Bosom*[1], *Antonio Dominguez-Alfaro*[1,2], *Matías L. Picchio*[2], *Daniele Mantione*[2], *David Mecerreyes*[2,3] *and George G. Malliaras*[1]

[1] Electrical Engineering Division, University of Cambridge, Cambridge, CB3 0FA, UK

[2] POLYMAT, University of the Basque Country UPV/EHU, Avda. Tolosa 72, Donostia-San Sebastián, Gipuzkoa 20018, Spain

[3] IKERBASQUE, Basque Foundation for Science, 48009, Bilbao, Spain

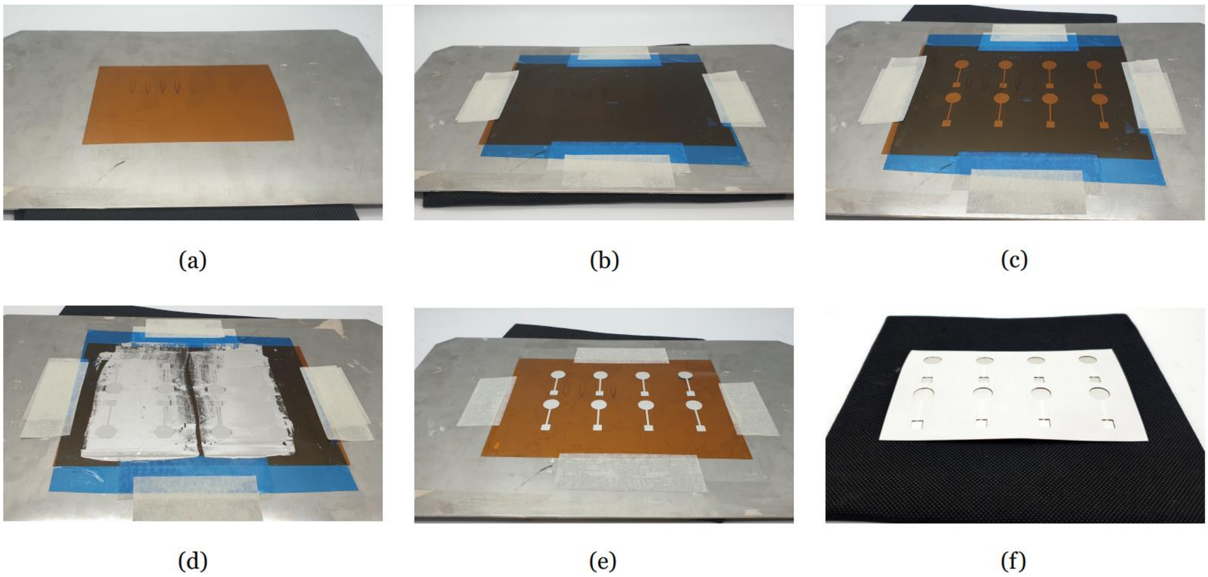

FIGURE S1 Electrode fabrication process. Kapton is employed as substrate (a), silicone release agent-free tape is attached to it (b) and is laser cut following a given electrode pattern (c), Ag nanoparticle paste is screen-printed over the tape mask (d), the tape is removed leaving a clean Kapton substrate with Ag electrodes and wires (e) and doubled-sided clinical tape is attached to the structure (f)

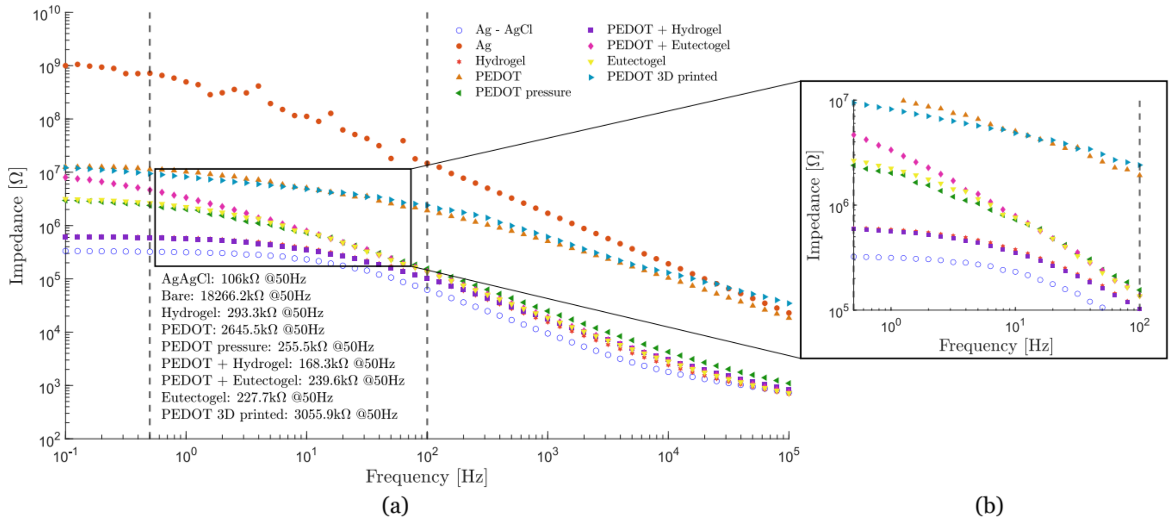

FIGURE S2 (a) Average impedance profile exhibited by single electrodes with different coating material as a function of frequency and (b) same diagram zoomed to the spectral region of interest for ECG measurements. Average impedance values at 50Hz are displayed

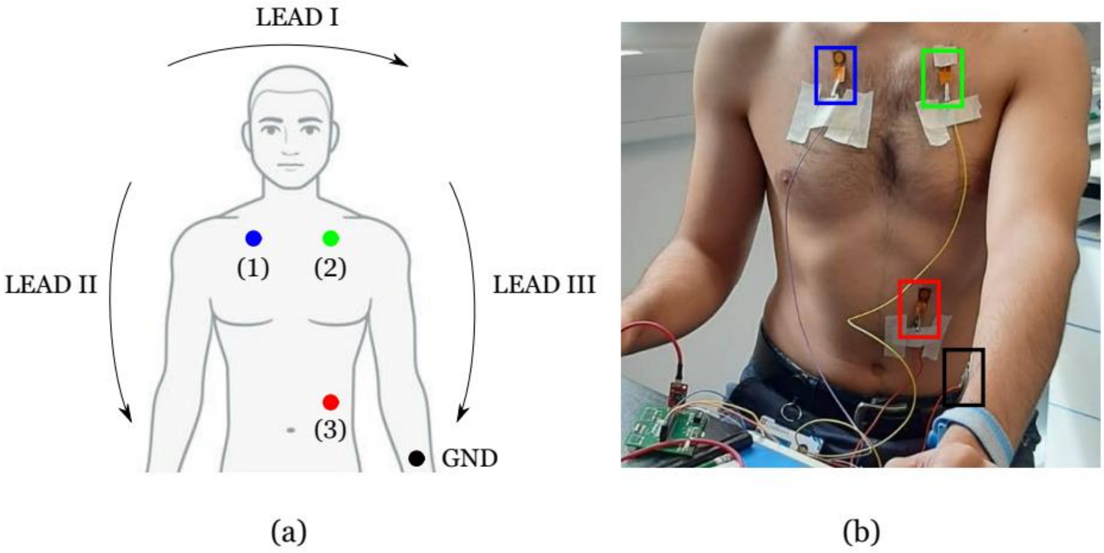

FIGURE S3 Conventional three electrode configuration. (a) Theoretical diagram, (b) real setup. GND indicates ground or reference electrode

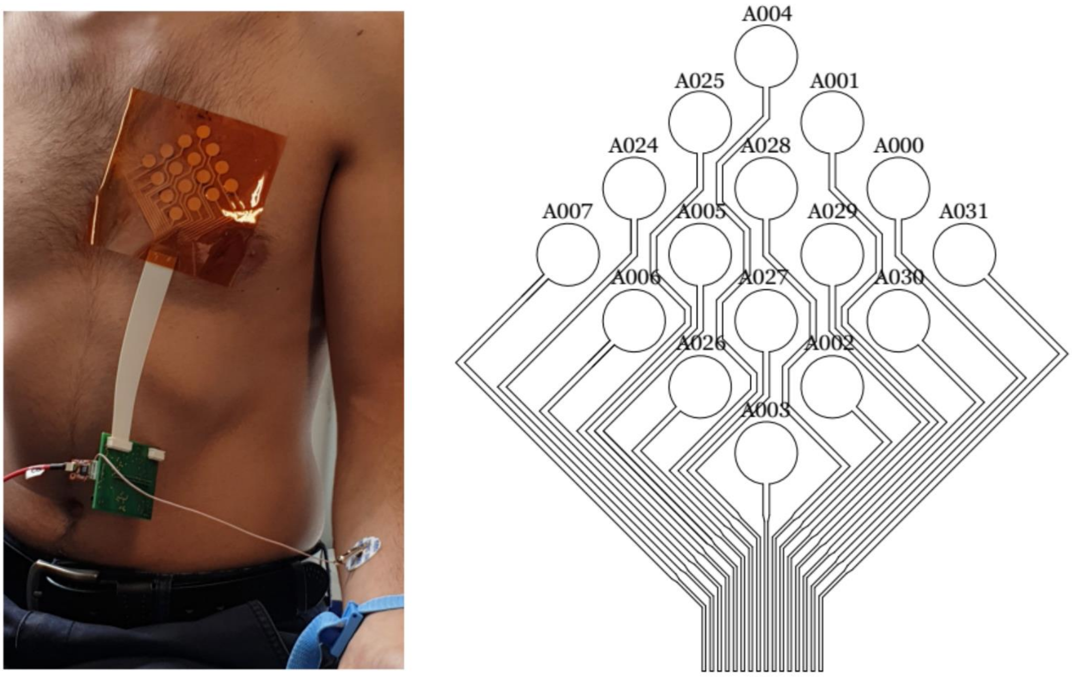

(a) (b)

FIGURE S4 (a) ECG electrode array physical setup and (b) respective mapping between electrode positioning and recording device channels

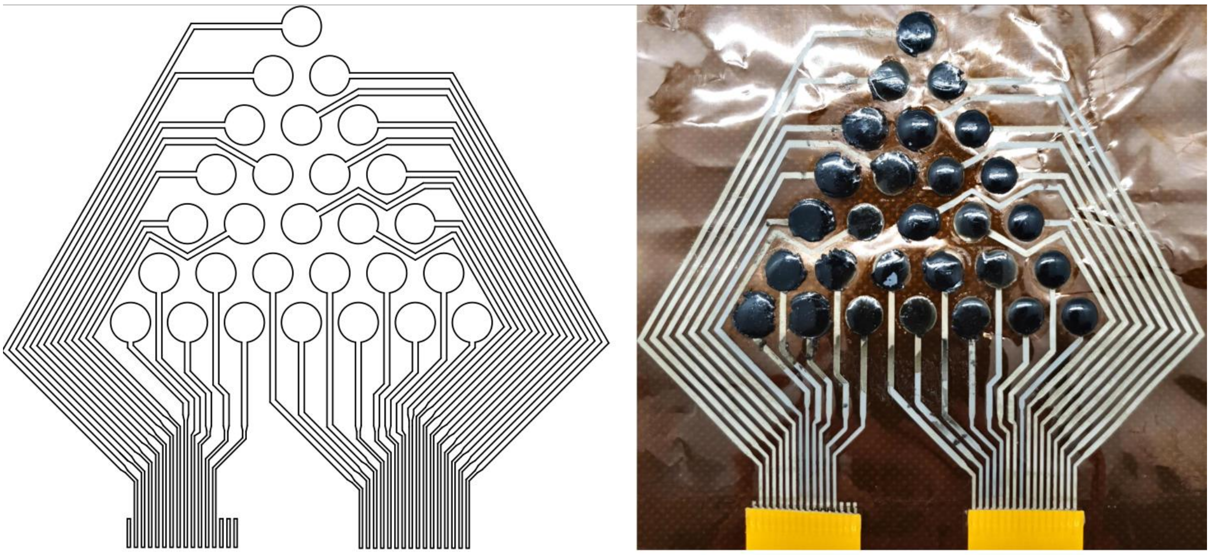

(a) (b)

FIGURE S5 (a) Triangular electrode array CAD design and (b) fabricated device

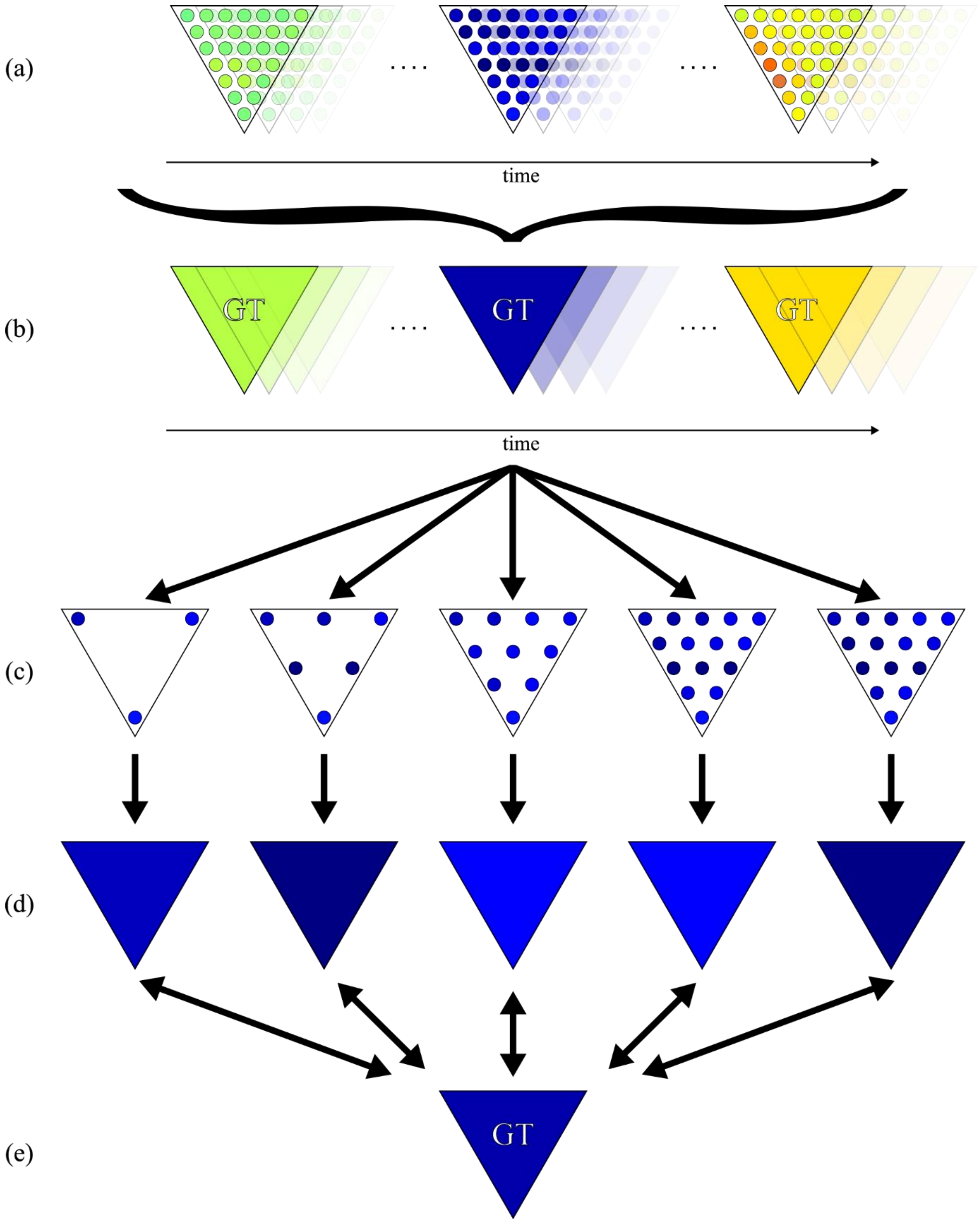

FIGURE S6 RMS error calculations of BSPM frames with different IED (a) BSPM frames are captured over time with a 28 electrode array with an IED of 10mm, (b) the frames are interpolated using the spline method to generate GT images, (c) the GT images are undersampled into frames with different IED, (d) each new frame is interpolated separately producing new images and (e) the images are compared with the GT image to yield RMS error values
